# Chicago Medical Society

**Published:** 1887-06

**Authors:** 


### Society Reports.

### THE CHICAGO MEDICAL SOCIETY.

[Official Report.]

Stated Meeting, March 21, 1887.

The President, Edmund J. Doering, M. D., in the chair.

Dr. James I. Tucker reported a case of

### HERPES OMOBRACHIALIS.

This class of skin diseases belongs to the neurological group. J. P., aged 36, had for many years been an invalid. In his youth he had been sexually intemperate and had used liquor to excess. He was of a family, every member of which had been afflicted with some form of nervous disorder. When he came under the author's care five years ago, he was emaciated, anæmic and suffering from profuse diarrhœa. He had pursued the vocation of proof-reader until six months previous, when he broke down. His habits were and had been for sometime past good in every respect. His principal trouble was a cough of deep, sepulchral sound, which gave him no rest night or day. No mucus or other foreign matter was expectorated. On the 3rd of December there was a neuralgic pain in the right shoulder ; on the next day an eruption appeared there attended with an almost insufferable sense of heat. The vascular eruption was fully developed by the 9th and occupied the shoulder and outward aspect of the arm, following the branch of the brachial plexus and terminating in an immense boil on the ball of the thumb. These vesicles passed through the various stages of vascular irritation, inflammation and resolution, and had entirely disappeared by December 15th ; and more remarkable still, the chronic cough disappeared altogether. To relieve the pain and burning sensation, I used hot fomentations and oleate of cocaine and ointment of glycerine. It was of no use to employ opiates, for although the patient was nervous and sleepless, morphia had no effect upon him. He took 240 grains of chloral hydrate of his own accord at a single dose without any other effect than to make him slightly delirious. The question arises, what may have been the relation between the cough and the eruption, and why did the cough cease on the disappearance of the eruption ?

Dr. Joseph Zeisler, in opening the discussion, thought cases of herpes zoster were comparatively rare here; in the last two years he had met with only one, affecting the fourth and fifth intercostal spaces of the right side. Although physiological and anatomical examinations have not yet proven the existence of true trophic fibres in the nerves, there can be no doubt that zoster must be considered as a trophoneurotic disease. The case was especially interesting from the possibility of syphilis being the cause of the disorder and from the large area involved.

Dr. Tucker said that one point in reference to zoster, especially zoster frontalis, is that chronic bronchitis is sure to follow it, and life generally terminates in that way.

Dr. Charles Warrington Earle asked if the author had not found that these cases almost always require the same tonic treatment that neuralgias do, viz., the exhibition of quinine and iron, and a good generous diet, before a cure can be effected.

Dr. Tucker replied that all the patients he had seen suffering from the disease have been depleted, and certainly the remedies just mentioned would be needed.

Dr. E. L. Holmes reported a case of

### CHANCRE OF THE EYELID.

Cases of chancre of the lids are of interest, not so much on account of their rareness as of the difficulty in establishing a certain and early diagnosis, and the fact that suspicion even may not be awakened till quite late in the development. It is many years, probably twenty, since I have seen a case of primary specific sore on the genital organs. If I remember correctly what

I formerly observed and what I learned from competent authorities, it is undoubtedly true that even an ulcer on the genital organs cannot always be easily diagnosticated. The swelling of the preauricular glands is quite often the result of simple abscess of the lids, especially if it is situated near the external angle. When, however, a circumscribed disease of the lids is accompanied by multiple preauricular and submaxillary glandular enlargement, suspicion becomes almost certainty. This has been the fact in four of the five cases which I have seen. In one case there was not sufficient swelling of the glands to attract either my attention or that of the patient.

Some months ago I mentioned in this Society the case of a laborer in a rolling mill who received a severe burn on the side of the body from a "flash" and on the left upper lid from a drop of the molten slag. This small round burn became infected from some source unknown to the patient, and after some time presented the appearance of a true chancre with glandular swellings and characteristic eruptions. No evidence of a lesion could be found on the genital organs. Every symptom disappeared under energetic mercurial treatment.

Very recently Mr. P. M., 45 years of age, married, also a workman in a rolling mill, received a "flash," a portion of which struck the left upper lid and inflicted quite a severe burn of the integument. The conjunctiva was also slightly burned. At the end of a week the patient came to me with the lids much swollen, red and tender, with a hard, dry, thin and red incrustation as one sees on a superficial abrasion of the skin. The patient stated that he had rubbed the lids several times during the week with the back of his hand, which was more or less covered with grime and sand. He did this to relieve the irritation of the eye. There was nothing in the appearance of the lids or of the eye to excite my suspicion of any complication. For ten days I treated the eye with mild lotions and ointments without the slightest benefit. On the appearance of preauricular and submaxillary swelling, I referred the patient to Dr. Hyde. I suspected the true nature of the case, although there was still no trace of ulceration on the lid. Prof. Hyde could simply suspect, but preferred to delay definite opinion for a few days. In a week or more the characteristic eruption appeared with increased severity of the local symptoms. Large doses of blue mass and iodide of potassium relieved all the symptoms, local as well as general. The eye assumed its normal appearance in about three weeks.

Dr. F. C. Hotz said that as the author wished the discussion to be conducted on the line of experience of general practitioners, not specialists, he hardly felt that he was the right person to open it. As the author states these primary syphilitic ulcerations, chancres of the eyelid, are of very rare occurrence, and in a long experience of private and hospital practice in this country and Europe he could remember but one case coming under his observation of a primary or syphilitic sore, and it was located on the conjunctival surface of the upper lid, a most curious place for the infection. A patient came to him about two years ago with the lid tender and very much swollen, and on turning it he saw a large ulcer with all the typical appearance of chancre of prepuce. There was no doubt of its syphilitic character, but it puzzled him as to how the infection could have taken place there. On questioning the patient he learned that four or five weeks previously he got a little cinder or something in his eye and his room-mate turned the upper lid, saw the foreign substance and removed it with his tongue. Further questioning brought out the fact that this man was syphilitic.

Dr. Joseph Zeisler mentioned the possibility of contamination by kissing and by instruments. The most peculiar cases of chancre in regard to the locality had been related by Deloir in his "Decours sur la Syphilis," some of which could only be explained by certain French habits.

Dr. William T. Belfield said, in reference to one point raised by Dr. Holmes, viz., infection without ulceration, he thought it was a well-known fact that it has frequently occurred. There is nothing about syphilitic infection that implies ulceration. The morbid material is introduced into the skin and occasions a certain amount of inflammation at the point of infection. Whether that results in ulceration depends upon the nutrition the parts receive; if they are well nourished there need be no ulcer, but as a rule some of the cells that are formed die and ulceration is the result. Occasionally the inflammation is so slight that neither ulceration nor even induration is produced locally, since syphilis is undeniably inoculated—in exceptional cases—without the production of any features of the hard chancre. Nor is the syphilitic the only virus whose local effects are thus variable; thus, the virus of charbon, or "wool sorters' disease," while usually producing a malignant pustule at the point of inoculation, may be communicated without any local ulceration—indeed, by simple inhalation.

Dr. A. K. Steele read a paper entitled

INTUSSUSCEPTION IN INFANTS.

Intussusception or invagination of the upper into the lower part of the intestine embraces three layers of the bowel which include all the coats of the intestines. The diagnosis of intussusception in an infant is usually an easy matter; a baby previously in good health is taken with sudden violent vomiting, with loud cries and evidence of abdominal pain and uneasiness occurring at frequent irregular intervals, accompanied with severe straining and the passage at first of fecal matter, then a mucus tinged with blood, and later of blood alone in considerable quantities. Thirst, anxious expression and collapse are marked symptoms. A distinct sausage-shaped tumor can frequently be felt at the seat of the obstruction in the abdomen.

Treatment may be divided into medicinal, mechanical and operative. The medicinal plan is palliative, but rarely curative.

The author described the details of the mechanical plan of treatment as follows: Having clearly established a diagnosis of intussusception and previously given an opiate to quiet all peristalsis, I anæsthetize the child with chloroform and then carry a rectal tube or large catheter about six inches within the anus. Attached to this tube I have a flexible rubber tube two or three feet long attached to a small glass funnel. Into this funnel I pour alternately solutions of soda bi-carbonate and tartaric acid, thus generating carbonic acid gas and rapidly and safely inflating the large intestine. Three drachms of each will generate enough gas to completely distend the colon of a child. If this pressure is not sufficient to relieve the obstructions, I attach an ordinary double bulb syringe and inflate with air about as much as I think the bowel will stand, while the abdomen is being carefully manipulated by an assistant. I patiently, persistently and repeatedly employ these measures, coupled with massage, abdominal taxis, inversion of child, etc., from twenty-four to forty-eight hours, when, if there is no relief, I advise laparotomy. In case the obstruction is at the ileo-cæcal valve and is irreducible and an artificial anus cannot be readily formed, an ileo-colotony or implantation of the divided ileum above the obstruction into a slit-like orifice in the colon below the obstruction would be justifiable procedure. Senn's recent experiments on animals prove that it can be done with success. Several cases were reported which had been successfully treated by the mechanical method.

Dr. C. W. Earle, in opening the discussion, said that Dr. Steele had presented the symptoms of this terrible difficulty so fully that there is little more to be said. He was glad that the author dwelt upon the symptom of constriction of part of the bowel, which is probably one of the chief causes for this accident. He must take issue with him in regard to the ease with which he diagnosticates some of these cases. Dr. Steele's experience has been very different from his own when he is able to say that the diagnosis of intussusception in a child is made out without much difficulty. In a number of cases which he has seen there has been an absence of all the symptoms which the author had narrated. He remembered a case which occurred several years ago in an outside village, in which he had made the post-mortem examination and found a very distinct intussusception. In that case we could get none of the symptoms Dr. Steele has spoken of; there was no tenesmus, no bloody discharge, no protrusion of the invaginated portion of the intestine, no sausage-shaped tumor of the abdomen, and yet the child had intussusception, probably from eating a large amount of cherries, as a number of cherry pits were found in the bowel at the autopsy.

As a medical man it might be expected that he would oppose a surgical procedure, but the medical treatment of this difficulty is so absolutely hopeless that it seems to him if laparotomy offers anything at all we should be ready to accept any procedure the surgeons have to offer. However, the results of Dr. Steele's process of treatment by inversion and shaking and filling the bowel are really better than those of surgeons up to the present time. It is a well-known fact that laparotomy for the relief of intussusception is followed by greater mortality than laparotomy for any other cause. But if the diagnosis is well made out, and particularly if the pulse is rapid and the child shows profound symptoms which point unmistakably to this difficulty, it seems to me we are hardly justified in waiting three days; he thought at the end of twenty-four hours we should cease to try the ordinary methods and proceed to do the operation.

Dr. F. E. Waxham said he agreed with Dr. Earle in regard to the difficulty of making the diagnosis in cases of intussusception, especially in infants. In many cases he thought the diagnosis very difficult indeed, and sometimes a certain and absolute diagnosis is almost impossible. This is true for the reason that all the symptoms that have been enumerated as characteristic of the disease are not present in every case; the tumor is as frequently absent as present; if a small amount of intestine is involved in the intussusception it is almost impossible to detect it. Again, the bloody discharges are frequently absent, and the protrusion of the intestine from the anus, which is given as a prominent symptom, is often absent. Indeed, in many cases the incessant vomiting, obstinate constipation and the tenesmus are the only symptoms. He would refer to the differential diagnosis of intussusception; fecal impaction occasionally presents symptoms analogous to intussusception. Impaction is rare in infancy, but is more frequently observed in the adult and in the older children. It may give rise to a tumor, vomiting, tenesmus, tympanitis and even protrusion of the intestine from the anus. These symptoms closely resemble those of intussusception, but in impaction the vomiting is not usually as obstinate, and large, copious enemas will cause the disappearance of the impaction. Again, in impaction we do not get the rapid and great prostration that is observed in intussusception. Cholera infantum may be mistaken for intussusception, for we have the same rapid and great prostration and collapse; we have the incessant vomiting, but in cholera infantum we have the large, copious, watery passages instead of the obstinate constipation followed by the bloody discharges. Dysentery presents symptoms closely resembling intussusception in many cases; we have vomiting, tenesmus, frequent straining, bloody passages and not unfrequently protrusion of the intestine from the anus, but in dysentery the vomiting is not so obstinate nor the prostration so great. Typhilitis may present symptoms very closely resembling intussusception; as the result of inflammation at the ileo-cæcal valve, we will have vomiting, intestinal pain, tympanitis, obstinate constipation, which it is impossible oftentimes to overcome. The diagnosis in these cases is very difficult, and I know of one case of typhilitis where several surgeons thought seriously of operating for a suspected intussusception. But in typhilitis the tumor, which results from inflammation and infiltration, is in the right side, while in intussusception the tumor when observed is in the left iliac region or in the transverse colon, for although the intussusception takes place at the ileo-cæcal valve, if the tumor is large enough to be observed, the smaller intestine rolls into the larger until the tumor appears in the left side instead of the right. In typhilitis we have a history of acute inflammation; not so in case of intussusception. Peritonitis will also frequently give rise to symptoms resembling those of intussusception; we have frequent vomiting, obstinate constipation, abdominal pain and tympanitis. He remembered a case that was treated for several days for intussusception, and not until the administration of morphia and lime water was the vomiting overcome and a correct diagnosis made. The vomiting could not have been relieved by medication in case of intussusception. In peritonitis we usually have the gradual onset of the disease, more sudden in case of intussusception. In peritonitis we have the symptoms of acute inflammation existing for several days before we get symptoms of obstruction. In intussusception the symptoms of obstruction and shock appear early.

In peritonitis, although the constipation is often obstinate, yet the vomiting is not as frequent and uncontrollable as in intussusception, nor do we have the rapid prostration in the former disease that is so characteristic of the latter. There are other forms of obstruction, such as the twisting of the intestine upon itself, pressure upon the intestine by a band of lymph, or strangulation of the intestine in a congenital opening in the diaphragm, any of which will give rise to symptoms exactly corresponding to intussusception. In regard to the treatment little remains to be said. Not infrequently, when an intussusception is suspected, the cases are treated with physic, castor oil, croton oil or quicksilver in order to prove the diagnosis. This treatment should be mentioned only to be condemned. No remedies should be given that will increase the peristaltic contraction, but opiates given to prevent it. Quicksilver may be used with benefit in those cases where the invagination is low down in the bowel, or where the intussusception is of such extent that it reaches into the lower bowel, or perhaps near the anus. In such cases the child should be inverted and the quicksilver used per rectum, and the pressure and weight of the quicksilver will assist greatly in the restoration of the invaginated mass. He fully agreed in the recommendation of laparotomy in cases where mechanical measures fail. When we have faithfully but unsuccessfully tried medicinal and mechanical measures, he thought that we should resort to laparotomy, providing a positive diagnosis can be made, and we should not delay too long, for the longer the operation is delayed the less the chances are of recovery.

Dr. A. E. Hoadley wished to make one suggestion as to the surgical treatment of these cases. The author, on going into the surgical treatment thoroughly, had left the idea, he thought, that about all that can be done for cases of acute invagination of the intestine is to unfold the invaginated intestine, and if this cannot be done, it must be excised and an artificial anus made. Of course he does not advise the resection of the invagination, but advises an artificial anus as a temporary means. Now, where this invagination is acute it obstructs the lumen of the intestine, and all the urgent symptoms are consequent on the obstruction of the bowels, not that the tissues are liable to ulceration or sloughing, provided that the tension above the invagination is promptly relieved. If the tension above the stricture is relieved the tissues at the stricture can take care of themselves; the strangulation will be immediately relieved in a measure, so that the circulation can be carried on at the seat of invagination. Relief of tension controls peristalsis and no further invagination will take place; congestion will be relieved and the tissues saved. There has been an operation suggested for that purpose which is applicable in all cases where to unfold the invagination is more dangerous than to leave it alone or excise and establish an artificial anus. The operation is to bring that portion of bowel above the invagination to that immediately below and there make an opening in each, stitch the openings together, and thus form an outlet for the distended intestine above. In case the invaginated portion loses its vitality there is no objection to its sloughing, as the slough can readily pass down, as the gut is not closed above. In his own practice he had seen two cases of recovery from intussusception, in which there was no doubt about the diagnosis; in one case a sausage-shaped tumor could be felt across the median line, with all the acute symptoms except, perhaps, the bloody discharges. The invaginations in these cases were of the colon, and not ileo-cæcal, and the lumen of the gut was not completely closed. There was no particular distension, but there was a large and painful tumor, with tenesmus and diarrhœa. In one case, in which the diagnosis was confirmed by several physicians, the tumor remained for more than a year, and at any time, if pressure was made over it, there would be tenesmus of the bowel, yet the child, who is now 8 years old, has fully recovered. He has a patient now under treatment in whom two and a half months ago there was probably intussusception of the colon. A painful tumor presented on the left side, which could be plainly felt from day to day, associated with all the irritable symptoms of the bowels, but the invagination did not occlude the lumen of the bowel; therefore the most distressing symptoms were absent. This tumor has gradually grown smaller, less tender, less painful, and the child is now about free from the irritation caused by that invagination.

Dr. J. Frank asked if, when the invagination is situated above the ileo-cæcal valve, water injected would pass the valve itself? He hardly thought the small amounts the author speaks of having injected would have reduced the invagination. He was not opposed to giving cathartics in cases where they might do good, and thought sometimes they do not do as much harm as an operation. The intestines are held up by mesenteries, and if the principal part of the intestine cannot invaginate itself where it is held fast, it cannot come down. A cathartic which produces a peristalsis is always towards the rectum, and if the part is held up by the mesentery, the peristaltic motion will not bring it down any further, and yet will relieve the intussusception. He thought many cases are cured by cathartics. He had a case sometime ago where the child was pulseless and cold, the eyes turned on the sockets, there was tenesmus and vomiting. After trying nearly everything an enormous dose of calomel was given, and as soon as there was a movement of the bowels the child was all right.

Dr. J. E. Colburn said that some years ago he had an interesting case; in the early morning he was called to see a child whom he found breathing with great difficulty and presenting many symptoms of acute bronchitis. He listened carefully and thought he located the region of the disturbance. Leaving some remedies he went away, promising to call late in the afternoon. During his absence the family became alarmed at a change of symptoms and called in another physician, who assured them that he was mistaken; that the child had acute gastritis. When he went back in the evening the child was purging and passing blood. He made an examination and found a tumor corresponding to the tumor described to-night. He gave a grave prognosis, left directions for the use of some remedies, and promised to see the child in the morning if it was still alive. The next morning when he called the mother told him the child was better, and showed him a chicken's foot and leg that it had swallowed.

Dr. Earle asked Dr. Steele to give the statistics in regard to the use of chloroform and ether with children.

Dr. Steele, in closing the discussion, said, in regard to Dr. Earle's query as to the ease of making the diagnosis: I would say that the diagnosis is not always an easy matter, and I do not wish to convey that impression. In the first case I reported the diagnosis was easy because the case presented the symptoms laid down in the books as nearly as could be; there was vomiting, tenesmus, bloody stools, absolute constipation and a lozenge-shaped tumor, but that is the only case of the four reported in which we had all the symptoms; in two there were no tumors at all; in one the tumor was indistinct and irregular in outline. There was no pain in that case. In none of the cases was the diagnosis verified by a post-mortem. There is an element of doubt as regards the diagnosis, but it had been formed in each case after careful examination by competent physicians and was considered fairly certain. In regard to the relative safety of ether and chloroform in children, I cannot give the statistics, but the administration of chloroform to children is a very safe procedure as compared with its administration to adults. In regard to the differential diagnosis, Dr. Waxham presented all the points very fully. In a paper of this character I did not wish to go into that subject. In regard to the operation of cutting the bowel above and below the point of obstruction, that I alluded to as an operation having been done experimentally on animals with success by Dr. Senn, of Milwaukee, so far as I know there are no statistics showing that it has been done on man. The case of obstruction occurring in a child, with painful tumor, spoken of by Dr. Hoadley, was a case of chronic obstruction in which the lumen was not perfectly occluded, and those cases are not dangerous. So long as the lumen is not absolutely interfered with the processes of nutrition go on. Cases of chronic obstruction were not considered in the paper; I spoke only of acute obstruction due to intussusception. Water passes the ileo-cæcal valve by dilatation from over-distension. I have seen the experiment tried on animals; by opening the abdomen and forcibly injecting water the colon would become distended to its utmost capacity, and by-and-by the tissues would begin to yield, the valve would become incompetent and the water would pass. The quantity of water used for injections was smaller than if the obstruction had been higher up. The case of acute obstruction mentioned by Dr. Frank, that was relieved by a large dose of calomel, was probably acute enteritis, such a case as would have been relieved by bleeding many years ago. When we are called to these cases of acute intestinal obstruction, where there is a doubt as to its cause, we should take into consideration all the factors—the age of the child, the sex, the suddenness of the onset, the presence of a majority of symptoms that are said to be present in intussusception, and then make a thorough, complete and systematic palpation of the abdomen, aided, if necessary, by the distension of the colon with carbonic acid gas. By the alternate injection of an acid and alkaline solution into the rectum, you can distend the colon so that it can be mapped out perfectly, and you can determine the location of the obstruction.

---

SPONTANEOUS PURIFICATION OF RIVER WATER.—It is well known that rivers which in flowing through towns become charged with various organic substances by degrees get rid of these impurities as they flow to a considerable distance. This can only be due to the oxidation of these organic matters, and M. Emich, who has studied the causes of this oxidation, arrives at the following conclusions:

If impure water be left undisturbed, and in contact with atmospheric air, it is purified in the same time as it would be by being shaken in contact with the air. Ozone is not perceptibly quicker in action than ordinary air; if impure water be boiled and left in contact only with air deprived of germs by filtration through cotton, the organic matters will remain in it. If the deposit precipitated by water kept in contact with the air be mixed with water previously boiled, organic matter quickly disappears. The process of oxidation consists in the transformation of ammonia and its compounds into nitric acid and nitrogen. These facts show that the spontaneous purification of water containing germs results from the development of organisms which act in the same way as the nitric ferment discovered by Schloesing.—*British Medical Journal.*

